# Prognostic implications of microRNA-100 and its functional roles in human epithelial ovarian cancer

**DOI:** 10.3892/or.2012.1625

**Published:** 2012-01-11

**Authors:** DONG-XIAN PENG, MIN LUO, LI-WEN QIU, YUAN-LI HE, XUE-FENG WANG

**Affiliations:** 1 Department of Obstetrics and Gynecology, Zhujiang Hospital, Southern Medical University, Guangzhou 510282; 2 Department of Obstetrics and Gynecology, Guangzhou General Hospital of Guangzhou Military Command, Guangzhou 510010, P.R. China

**Keywords:** epithelial ovarian carcinoma, microRNA-100, TaqMan real-time RT-PCR, prognosis, overall survival, polo-like kinase 1

## Abstract

Dysregulation of microRNAs (miRNAs) has been found to be associated with a variety of diseases, including epithelial ovarian cancer (EOC). Recently, miR-100 was reported to be downregulated in human ovarian carcinoma, however, the clinical significance and functional roles of miR-100 expression in human EOC are unclear. TaqMan real-time quantitative RT-PCR assay was performed to detect the expression of miR-100 in 98 EOC tissues and 15 adjacent normal epithelial tissues. The relationship between miR-100 expression and clinicopathological factors in 98 EOC patients was statistically analyzed. The effect of miR-100 expression on patient survival was determined. Finally, the role of miR-100 in EOC cell growth and its possible mechanisms were analyzed with miR-100 precursor or inhibitor-transfected cells. We showed that the level of miR-100 was significantly lower in EOC tissues compared to adjacent normal tissues. Low miR-100 expression was found to be closely correlated with advanced FIGO stage, higher serum CA125 expression level and lymph node involvement. Also, low miR-100 expression was correlated with shorter overall survival of EOC patients, and multivariate analysis showed that the status of miR-100 expression was an independent predictor of overall survival in EOC. Additionally, miR-100 could affect the growth of EOC cells by post-transcriptionally regulating polo-like kinase 1 (PLK1) expression. Together, these results suggest that low miR-100 expression may be an independent poor prognostic factor and miR-100 can function as a tumor suppressor by targeting PLK1 in human EOCs.

## Introduction

Epithelial ovarian cancer has been reported to be the second most common gynecological cancer and accounts for nearly half of all deaths associated with gynecological pelvic malignancies (1). Although improvement of the quality of cytoreductive surgery as well as the development of novel drugs and new chemotherapy regimens for ovarian carcinoma therapy have been made, prognosis remains poor for as many as 50% of the patients who will experience recurrence and die of secondary disease within 5 years after diagnosis (2). Thus, it is needed to identify some novel molecular mechanisms involved in ovarian cancer initiation and development.

microRNA (miRNA) belongs to a class of endogenously expressed, non-coding small RNAs, which have emerged as key post-transcriptional regulators of gene expression (3). In mammals, miRNAs have been predicted to control the activity of approximately 30% of all protein-coding genes, and have been shown to participate in the regulation of almost every cellular process investigated so far (4,5). Recently, dysregulation of miRNAs has been found to be involved in a variety of human diseases including cancer (6,7). Up to date, many researches have studied miRNA expression in ovarian carcinoma by using various gene expression profiling approaches (8). By an integrative genomic approach, Zhang *et al* showed that out of 35 miRNAs deregulated between ovarian carcinoma and the normal controls (immortalized ovarian surface epithelial cells), 31 (88.6%) were downregulated in cancer compared to non-cancer tissues (9). By miRNA microarrays, 36 miRNAs were found to be deregulated between normal ovarian cells and epithelial ovarian tumors, with miR-199a^*^, miR-214, miR-200a, and miR-100 being the most highly differentially expressed candidates (10). In particular, miR-100 was found to be downregulated in 76% of tumors. In another research, a subset of 37 miRNAs was found to be overexpressed in all epithelial ovarian cancer subtypes and 21 were underexpressed and the validated downregulated genes included miR-100, miR-210, miR-22 and miR-222 (11). Although miR-100 was reported to be significantly downregulated in EOC tissues, the correlation of miR-100 expression with clinicopathological factors or prognosis of patients with EOC and its functional roles in EOC remain unclear.

In this study, our aim was to determine the expression of miR-100 in 98 EOC tissues and corresponding adjacent normal epithelial tissues. Then, the clinicopathological or prognostic significance of miR-100 expression in human EOCs was statistically analyzed. Next, a miR-100 inhibitor or precusor was transiently transfected into EOC cell lines, and the effect of miR-100 expression on the growth of EOC cells was analyzed. Finally, whether polo-like kinase 1 (PLK1) was a target regulated by miR-100 expression was also determined.

## Materials and methods

### Patients and tissue samples

A total of 98 fresh surgical tissue samples of EOC and 15 adjacent normal epithelial tissues were collected at the Jiangsu Province Hospital between 2002 and 2004, after informed consent had been obtained. An independent pathologist assigned histopathology and tumor grade according to International Federation of Gynecology and Obstetrics (FIGO) criteria. A gynecologic oncologist reviewed tumor stage and residual disease. Normal tissues were obtained from tumor-free participants that had undergone oophorectomy. Namely, they underwent surgery for a total hysterectomy, bilateral salpingo-oophorectomy, partial omentectomy, appendectomy, and pelvic and para-aortic lymphadenectomies. The clinicopathological variables of patients were shown in Table I. All tissue samples were snap-frozen in liquid nitrogen, which were transferred to 500 ml TRIzol solution (Invitrogen, Carlsbad, CA, USA) immediately after harvesting in order to avoid mRNA degradation. The samples were stored in a biobank at −80°C until processed.

### Cell culture

The EOC cell line (SKOV-3) was purchased from the Shanghai Institute of Cell Biology (Shanghai, China). All cell lines were cultured in RPMI-1640 (Gibco-BRL) medium supplemented with 10% fetal bovine serum (FBS), 100 U/ml penicillin, and 100 μg/ml streptomycin in humidified air at 37°C with 5% CO_2_.

### TaqMan real-time quantitative RT-PCR assay

Real-time qRT-PCR analysis of mature miRNA was performed using the TaqMan microRNA Assay kit (Applied Biosystems, Foster City, CA) as previously described (12).

### Western blot assay

Total cellular protein extract was isolated from harvested cells, protein concentration was determined, and western blot analysis was carried out as previously described (13). The antibodies used were anti-PLK1 and anti-GAPDH (Santa Cruz Biotechnology, Inc., Santa Cruz, CA).

### Plasmid of construction

Two pairs of primers were used to generate PLK1 fragment based on the published PLK1 sequence (GenBank Accession no. NM_005030). The sequences of primers for the fragment were: sense 5′-ATGA GTGCTGCAGTGACTGCAGGGAAG-3′ antisense 5′-AGC TATTAGGAGGCCTTGAGACGG-3′. Reverse transcription was carried out using the EOC cell RNA as a template. Reverse transcription PCR products were cloned into the pcDNA3.1 vector (Invitrogen) with the sequences and orientations confirmed from both ends to generate the recombinant vector pcDNA/PLK1.

### Transfection of oligonucleotides or plasmids

Molecules of dsRNAs that mimic endogenous hsa-miR-100 (pre-miR-100) and single-strand miR-100 inhibitor (anti-miR-100), designed to inhibit endogenous hsa-miR-100, were purchased from Ambion (Austin, TX, USA). The siRNAs were designed and synthesized by GenePharma (Shanghai, China). PLK1 siRNA (siRNA/PLK1) was synthesized as follows: sense 5′-AAGGGCGGCUUUGCCAAGUGC-3′; negative control siRNA (siRNA/control) were synthesized as follows: sense 5′-UUCUCCGAACGUGUCACGUTT-3′. siRNAs, miRNAs and plasmid transfections were performed using Lipofectamine 2000 (Invitrogen). In brief, cells were plated in 6-well plates to 50% confluence. For each well, 5 μl siRNA or miRNA (20 μM) or 5 μg plasmid was added into 250 μl Opti-MEM medium, 5 μl of Lipofectamine 2000 into 250 μl Opti-MEM medium, and then mixed with siRNA or miRNA or plasmid with Lipofectamine 2000. The mixture was added to the cells and incubated for 6 h before replacing the medium.

### Cell proliferation assay

Cell proliferation was determined by the MTT assay. In brief, the cells were transfected with miRNA mimics or inhibitors after 48 h. The MTT solution in PBS was added to attain a final concentration of 0.5 mg/ml, and incubation was continued for 4 h. Finally, an equal volume of a lysis buffer containing 50% dimethylformamide and 20% sodium dodecyl sulfate (pH 4.8) was added. The mixtures were kept overnight and then the amount of MTT formazan present was quantified by determining its absorbance at 490 nm using an ELISA plate reader (Wallac).

### Luciferase reporter assays

The 3′-UTR of human PLK1 (GenBank Accession no. NM_005030) was amplified from human genomic DNA and individually cloned into the pGL3-Basic vector (Ambion) by directional cloning. Seed regions were mutated to remove all complementarity to nucleotides 2–8 of miR-100 by using the QuickChangeXL mutagenesis kit (Stratagene, La Jolla, CA). HEK-293 cells were co-transfected with 0.4 mg of firefly luciferase reporter vector and 0.02 mg of the control vector containing *Renilla* luciferase, pRL-SV40 (Promega), using Lipofectamine 2000 (Invitrogen) in 24-well plates. Each transfection was carried out in four wells. For each well, 50 nM of pre-miR-100 (anti-miR-100) or a negative control pre-miR-NC (anti-miR-100) was co-transfected with the reporter constructs. Luciferase assays were performed 24 h after transfection using the Dual Luciferase Reporter Assay system (Promega). Firefly luciferase activity was normalized to *Renilla* luciferase activity.

### Statistical analysis

Statistical analysis was performed using SPSS 17.0. For continuous variables, data are expressed as mean ± standard deviation (SD). The relationship between miR-100 expression and clinicopathological factors was analyzed using the Student’s t-test. Survival probabilities were calculated by the product limit method of Kaplan-Meier. Differences between the groups were tested using the log-rank test. The results were analyzed for the endpoint of 5 years overall survival (OS). Survival times of patients still alive were censored with the last follow-up date. The Cox proportional hazards regression model was used to assess the independence of different prognostic factors. It was considered significant when the P-value was <0.05.

## Results

### The expression of miR-100 is significantly downregulated in human EOC tissues

**Figure 1:**
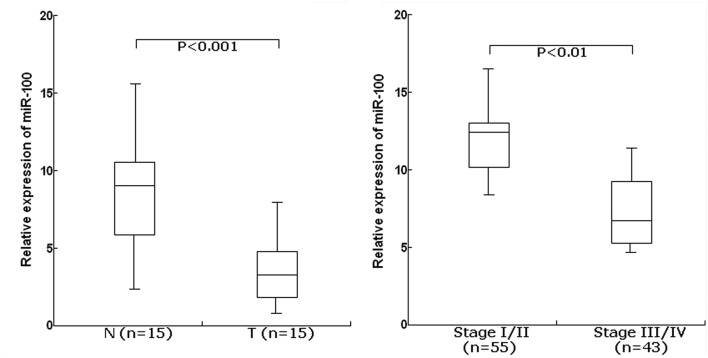
TaqMan real-time RT-PCR analysis of relative miR-100 expression in tissue samples. (A) Levels of miR-100 was significantly lower in EOC tissues (n=15) than in a adjacent normal tissue (n=15) (P<0.001). (B) Levels of miR-100 were confirmed to be lower in patients with FIGO stage III/IV (n=43) as compared to stage I/II (n=55). Statistical significance comparing the expression of miR-100 between EOC and adjacent normal tissue was calculated using the Wilcoxon signed-rank test.

The TaqMan real-time RT-PCR assay was performed to determine the expression of miR-100 in 15 cases of EOC and their matched normal tissues. For the EOC tissues, the mean level of miR-100 expression was 3.8 (range, 1.6–8.5). For the matched normal tissues, the mean level of miR-100 expression was 8.4 (range, 2.7–16.4). The level of miR-100 was significantly lower in EOC tissues than in adjacent normal tissues (P<0.001; Fig. 1A). Furthermore, the expression level of miR-100 was significantly lower in EOC patients with advanced clinical stage (III/IV) compared with those with clinical stage (I/II) (P<0.01; Fig. 1B). From these data, it was concluded that downregulation of miR-155 may play a critical role in ovarian tumorigenesis.

### Association of miR-100 expression with clinicopathological characteristics in EOC

Next, the clinicopathological significance of miR-100 expression in human EOC was analyzed. Patients with relative miR-100 expression levels in EOC tissues less than the median value of 0.14 formed the low expression group (n=50), while patients with relative miR-100 expression levels in EOC tissues ≥0.14 formed the high expression group (n=48). As shown in Table I, the low level of miR-100 expression was closely correlated with advanced FIGO stage, higher serum CA125 expression level and lymph node involvement (P=0.001, 0.001 and 0.014, respectively). However, there was no significant correlation between miR-100 expression and other clinicopathological variables of EOC patients including age, histological type, histological grade, residual tumor diameter and ascites (P=0.155, 0.486, 0.849, 0.366 and 0.279, respectively). Interestingly, we also found that the incidence of lymph node metastasis in EOC patients with low miR-100 level (58.0%) was significantly higher than that in patients with high miR-100 levels (33.3%).

### Association of miR-100 expression with prognosis of EOC patients

**Figure 2:**
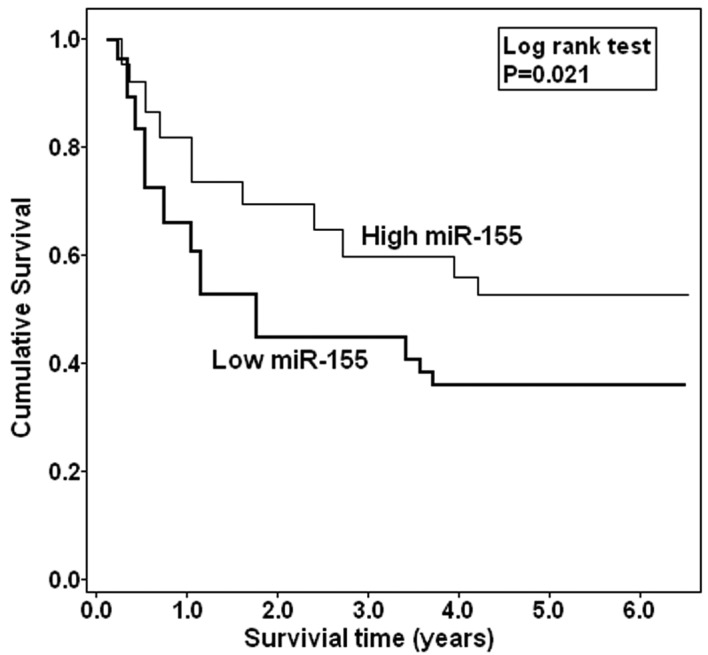
Survival curves of EOC patients according to the status of miR-100 expression. Patients with low miR-100 expression (n=50) showed significantly poorer prognosis than those with high miR-100 expression (n=48) (P=0.021; log-rank test).

Whether miR-100 expression could affect prognosis of patients was statistically analyzed. The Kaplan-Meier curve is shown in Fig. 2. Those EOC patients with low miR-100 expression were more likely to have a shorter overall survival (P=0.021), when compared to patients with high miR-100 expression. These data are further supported by the Cox regression analysis presented in Table II. By univariate analysis, the status of miR-100 expression was correlated with poor survival of EOC patients (P=0.038). Finally, a multivariate analysis with the Cox proportional hazards showed that the status of miR-100 expression, along with FIGO stage and lymph node involvement, was an independent predictor of overall survival in EOC (RR, 2.12; 95%CI, 1.88–3.41; P=0.008).

### Effect of miR-100 expression on in vitro proliferation of EOC cells

**Figure 3:**
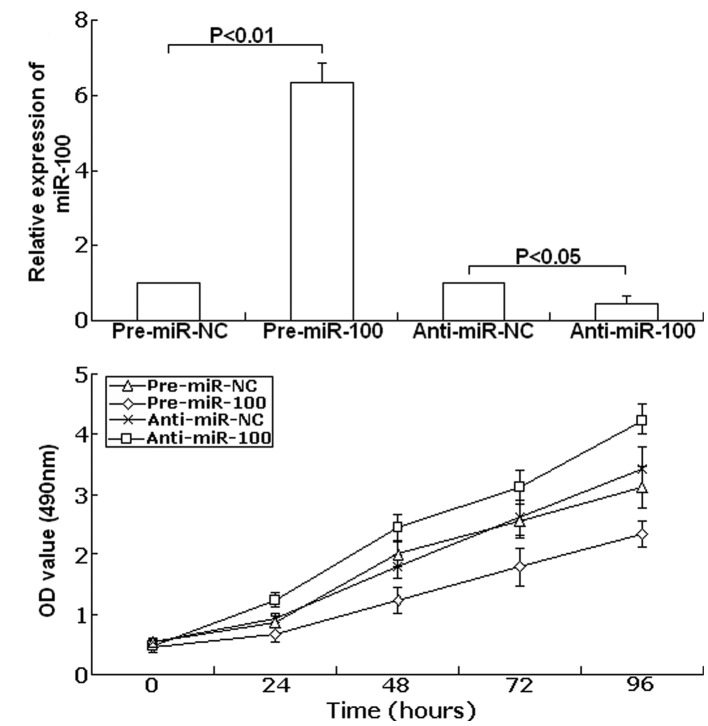
Effect of miR-100 expression on the growth of the EOC cell line (SKOV-3). (A) TaqMan real-time RT-PCR analysis of relative miR-100 expression in the miR-100 precusor (pre-miR-100) or inhibitor (anti-miR-100)-tranfected SKOV-3 cells. Pre-miR-NC or anti-miR-NC was used as a corresponding control. (B) MTT analysis of growth in SKOV-3 cells transfected with pre-miR-100 (pre-miR-NC) or anti-miR-100 (anti-miR-NC). Data are presented as the mean ± SD of three experiments.

In order to study the functional role of miR-100 in ovarian tumorigenesis, SKOV-3 cells were transfected with pre-miR-100, anti-miR-100 or scrambled precursor (pre-miR-NC)/antisense oligonucleotide control (anti-miR-NC). Forty-eight hours after transfection, TaqMan real-time RT-PCR assay was performed to examine the expression of miR-100. As shown in Fig. 3A, compared with precursor control, the expression of miR-100 showed up-regulation to 6.34-fold in the transfected group with pre-miR-100 (P<0.01). Meanwhile, compared with the antisense control, the expression of miR-100 was downregulated to 45.6% (P<0.05). Next, the cell viability was determined using the MTT assay. As shown in Fig. 3B, in SKOV-3 cells transfected with anti-miR-100 cell viability significantly increased compared with the anti-miR-NC-transfected cells. In contrast, upregulation of miR-100 led to decreased cell viability compared with the pre-miR-NC-transfected cells. These data show that cell viability could be inhibited by upregulation of miR-100, and could be promoted by downregulation of miR-100.

### Polo-like kinase 1 (PLK1) is a target of miR-100

**Figure 4:**
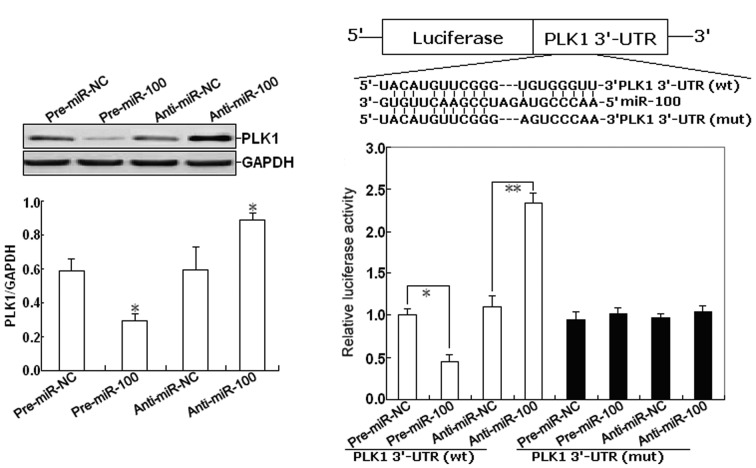
PLK1 is a target of miR-100. (A) Western blot analysis of PLK1 protein expression in SKOV-3 cells transfected with pre-miR-100 (pre-miR-NC) or anti-miR-100 (anti-miR-NC). (B) PLK1 3′-UTR and corresponding fragments were inserted into the region immediately downstream of the luciferase gene in pGL3-Basic vector and validated by DNA sequencing. The sequences of the predicted miR-100 binding sites within the PLK1 3′-UTR, including wild-type UTR or UTR segments containing mutant binding site are shown. (C) Detection of luciferase activity. For luciferase reporter assays in 6-well plates, 1-mg luciferase reporter plasmid containing either wild-type (wt) or mutant (mut) PLK1 3′-UTR, and 200 pmol of pre-miR-100, anti-miR-100, pre-miR-NC or anti-miR-100 were transfected. The parental luciferase plasmid was also transfected as a control. At 24 h after transfection, cells were assayed using the Luciferase Gene Reporter Assay kit. The data are presented as the mean ± SD of three experiments. ^*^P<0.05 or ^**^P<0.01.

In a previous study, PLK1 has been reported to be a miR-100 target in human nasopharyngeal cancer. However, whether PLK1 is a direct miR-100 target in EOC cells is unknown. To confirm this, we firstly determined the effect of miR-100 expression on the expression of PLK1 protein in EOC cells. As shown in Fig. 4A, western blot assay indicated that pre-miR-100 could induce the decreased expression of PLK1 protein, but anti-miR-100 could induce the increased expression of PLK1 protein in EOC cells (P<0.05). To further confirm the target specificity between miR-100 and its potential target, PLK1, we carried out a luciferase reporter assay with a vector containing the putative PLK1 3′-untranslated region (UTR) target site downstream of the luciferase reporter gene. The base pairing between miR-100 and the wild-type (wt) or mutant (mut) target site in the 3′-UTR of PLK1 mRNA is shown in Fig. 4B. Next, SKOV-3 cells were co-transfected with PLK1-3′-UTR luciferase reporter (wt-PLK1-3′-UTR) and pre-miR-100 or anti-miR-100. Transfections with control vector were performed in parallel. As shown in Fig. 4C, pre-miR-100 or anti-miR-100 significantly decreased or respectively increased the activity of the Luc-PLK1-3′-UTR reporter (P<0.01). However, transfection of the mut-PLK1-3′-UTR reporter did not affect reporter activity (P>0.05).

**Figure 5:**
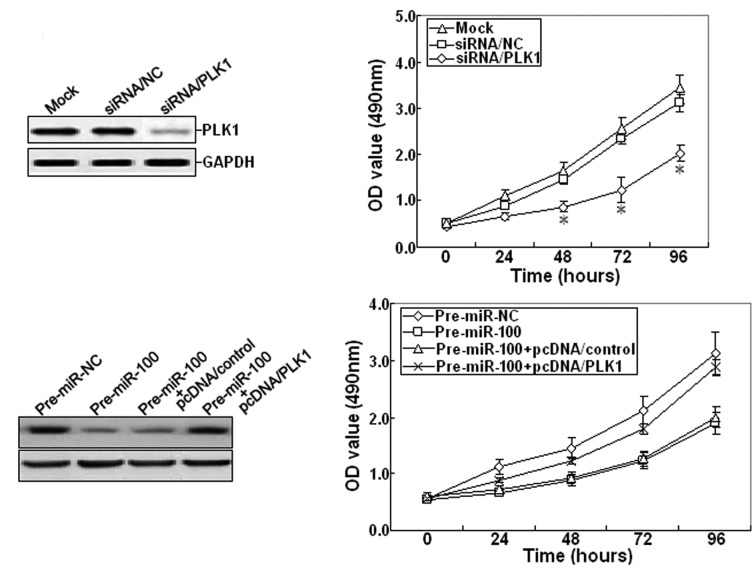
Functional analysis of PLK1 gene in SKOV-3 cells. (A) Western blot analysis of PLK1 protein expression in mock or siRNA/PLK1 (siRNA/control)-transfected SKOV-3 cells. (B) MTT analysis of cell growth in mock SKOV-3 or SKOV-3 cells transfected with siRNA/PLK1 or siRNA/control. (C) Western blot analysis of PLK1 protein expression in pre-miR-100 (or pre-miR-NC)-transfected SKOV-3 cells or co-transfected with pcDNA/PLK1 (or pcDNA/control). (D) MTT analysis of growth in pre-miR-100 (or pre-miR-NC)-transfected SKOV-3 cells or co-transfected with pcDNA/PLK1 (or pcDNA/control). The data are presented as the mean ± SD of three experiments. ^*^P<0.05.

Furthermore, 48 h after siRNA/PLK1 was transfected into SKOV-3 cells, RT-PCR and western blot assays showed that siRNA/PLK1 could significantly inhibit the expression of PLK1 mRNA and protein (Fig. 5A). The cell viability was also determined. The growth of siRNA/PLK1-transfected SKOV-3 cells was inhibited compared with mock or siRNA/control-transfected cells (Fig. 5B). Next, pcDNA/PLK1 vector and pre-miR-100 were co-transfected into the SKOV-3 cell line. Western blot analysis indicated that pcDNA/PLK1 could reverse the decreased expression of PLK1 protein induced by pre-miR-100 (Fig. 5C). Meanwhile, overexpression of PLK1 could also reverse the inhibitory growth induced by pre-miR-100 (Fig. 5D). Therefore, it was concluded that miR-100 may function as a tumor suppressor in EOC by targeting PLK1.

## Discussion

Recently, much research effort has been intensely focused on the roles of dysregulated miRNA expression in various human cancer types including human EOC (14,15). Many studies have shown that abberant miRNAs are asscociated with proliferation, apoptosis, metastasis, and chemoresistance of tumor cells (16–18). Thus, identification of the important miRNA expression signatures in EOC development and progression will be helpful to find potential diagnostic and prognostic markers for EOC diagnosis and treatment.

Up to date, there have been many published research studies about the correlation of miRNAs with EOC. Lu *et al* reported that hypermethylation of let-7a-3 in epithelial ovarian cancer was associated with low insulin-like growth factor-II expression and favorable prognosis (19). Additionally, the beneficial impact of the addition of paclitaxel on EOC survival has been significantly linked to let-7a levels, and it has been shown that miRNAs such as let-7a may be useful markers for the selection of chemotherapeutic agents in EOC management (20). Lou *et al* reported that microRNA-21 could promote the cell proliferation, invasion and migration abilities in ovarian epithelial carcinomas through inhibiting the expression of PTEN protein (21). In other studies, several other microRNAs have been reported to be associated with chemotherapy resistance, such as miR-214, miR-130a, miR-27a and miR-451 (22–24). Although miR-100 was reported to be downregulated in human EOC by other research groups, the clinicopathological or prognostic significance of miR-100 in EOC is still unknown. In nasopharyngeal cancer, underexpressed miR-100 was found to lead to PLK1 overexpression, which in turn contributes to NPC progression (25). However, in prostate cancer, it was reported that a high level of miR-100 was related to biochemical recurrence of localized prostate cancer in patients treated with radical prostatectomy (26). Recently, Zheng and his group reported that miR-100 could regulate cell differentiation and survival by targeting RBSP3, a phosphatase-like tumor suppressor in acute myeloid leukemia (27). From the above studies, it may be concluded that miRNA oncogenes and tumor suppressors show different patterns in different tumor types.

In the present study, we firstly showed that the mean level of miR-100 expression in EOC tissues was significantly higher than that in the matched normal tissues. Also, the expression level of miR-100 was significantly lower in EOC patients with advanced clinical stage (III/IV) compared with those with clinical stage I/II. Meanwhile, we showed that low miR-100 expression was closely correlated with advanced FIGO stage, high serum CA125 level and lymph node involvement. Furthermore, patients with low miR-100 expression showed poorer survival than those with high miR-100 expression. A multivariate analysis with the Cox proportional hazards showed that the status of miR-100 expression was an independent predictor of overall survival in EOC. Then, we analyzed the effect of miR-100 expression on the growth of EOC cells and its possible mechanisms. Overexpression of miR-100 could induce growth suppression in human EOC cells, while downregulation of miR-100 could promote growth of EOC cells. PLK1 is a serine/threonine kinase that functions to regulate many stages of mitosis (28). The overexpression of PLK1 has been found in many human cancers, including ovarian carcinoma (29). It has been reported that the overexpression of PLK1 could affect growth, apoptosis, metastasis and chemo-or radioresistance in human cancers (30,31). Weichert *et al* showed that PLK1 is a novel independent prognostic marker in ovarian carcinomas (29). Additionally, silencing of Chk1 and PLK1 could enhance radiation-or cisplatin-induced cytotoxicity in human ovarian cancer cells (32). In our study, we show that overexpression of miR-100 could inhibit the expression of PLK1 protein in EOC cells. So, it can be demonstrated that miR-100 negatively regulated PLK1 at the post-translational level, acting as a tumor suppressor in the EOC. *In vitro* luciferase assay further suggested that PLK1 is the target gene of miR-100. Importantly, siRNA-mediated downgulation of PLK1 could recapitulate the tumor suppressor function of miR-100, and overexpression of PLK1 could partly rescue the reduced cellular proliferation observed upon miR-100 upregulation in SKOV-3 cells, demonstrating that PLK1 is an important functional target of miR-100 in this model.

In conclusion, this study suggests that miR-100 is downregulated in human EOC and low miR-100 expression may be a poor prognostic factor. Also, miR-100 can significantly inhibit growth of EOC cells by targeting PLK1. Thus, this miR-100/PLK1 signaling pathway may provide therapeutic targets for human EOCs. This study has several limitations. Firstly, the number of tissue samples is small, and further investigation of a larger case population is needed to confirm the prognostic significance of miR-100 expression in EOC. Future investigations using patient samples are needed to further support the function of miR-100 in EOC.

## Figures and Tables

**Table I:** Association of miR-100 expression with clinicopathological variables of EOC patients.

	Low miR-100 expression (n=50)	High miR-100 expression (n=48)	
			
Variables	n (%)	n (%)	P-value
Age (years)			0.155
≥50	36 (72.0)	28 (58.3)	
<50	14 (28.0)	20 (41.7)	
Histological type			0.486
Serous	16 (32.0)	11 (22.9)	
Mucinous	21 (42.0)	20 (41.7)	
Others	13 (26.0)	17 (35.4)	
Histological grade			0.849
G1	25 (50.0)	22 (45.8)	
G2	10 (20.0)	9 (18.8)	
G3	15 (30.0)	17 (35.4)	
FIGO stage			0.001
I/II	14 (28.0)	29 (60.4)	
III/IV	36 (72.0)	19 (39.6)	
Residual tumor diameter (cm)			0.366
<1.0	20 (40.0)	15 (31.3)	
≥1.0	30 (60.0)	33 (68.7)	
Ascites			0.279
No	22 (44.0)	16 (33.3)	
Yes	28 (56.0)	32 (66.7)	
Serum CA125 level (U/l)			0.001
<5.0×10^5^	15 (30.0)	30 (62.5)	
≥5.0×10^5^	35 (70.0)	18 (37.5)	
Lymph node involvement			0.014
No	21 (42.0)	32 (66.7)	
Yes	29 (58.0)	16 (33.3)	

**Table II:** Univariate and multivariate analysis of prognostic variables by Cox regression analysis.

	Univariate analysis		Multivariate analysis	
		
Clinicopathological variables	RR (95% CI)	P-value	RR (95% CI)	P-value
Age (years) (≥50/<50)	1.41 (0.69–1.72)	0.215	1.56 (0.91–2.45)	0.098
Histological type (Serous/non-serious)	0.94 (0.81–1.08)	0.156	2.56 (0.89–3.12)	0.227
Histological grade (G1/G2+G3)	2.43 (1.68–2.94)	0.008	2.13 (0.75–2.70)	0.089
FIGO satge (III+IV/I+II)	3.08 (2.23–4.18)	0.016	1.69 (1.23–2.55)	0.005
Residual tumor (cm) (≥1.0/<1.0)	1.48 (0.77–1.82)	0.105	1.65 (0.87–1.93)	0.274
Ascites (Yes/no)	0.85 (0.71–1.16)	0.476	1.87 (0.63–2.63)	0.078
Serum CA125 (≥5.0×10^5^/<5.0×10^5^ U/l)	1.37 (0.82–1.79)	0.123	2.06 (0.90–3.12)	0.244
Lymph node involvement (Yes/no)	2.12 (1.59–2.34)	0.023	3.18 (2.18–4.03)	0.011
Expression of miR-100 (Low/high)	1.65 (1.16–2.73)	0.038	2.12 (1.88–3.41)	0.008

RR, relative ratio; 95% CI, 95% confidence interval.

## References

[1] Jemal A, Siegel R, Ward E, Hao Y, Xu J, Thun MJ (2009). Cancer statistics, 2009. CA Cancer J Clin.

[2] Armstrong DK (2002). Relapsed ovarian cancer: challenges and management strategies for a chronic disease. Oncologist.

[3] Bartel DP (2004). MicroRNAs: genomics, biogenesis, mechanism, and function. Cell.

[4] Huang Y, Shen XJ, Zou Q, Zhao QL (2010). Biological functions of microRNAs. Bioorg Khim.

[5] Huang Y, Shen XJ, Zou Q, Wang SP, Tang SM, Zhang GZ (2011). Biological functions of microRNAs: a review. J Physiol Biochem.

[6] Perera RJ, Ray A (2007). MicroRNAs in the search for understanding human diseases. BioDrugs.

[7] Calin GA, Croce CM (2006). MicroRNA signatures in human cancers. Nat Rev Cancer.

[8] Dahiya N, Morin PJ (2010). MicroRNAs in ovarian carcinomas. Endocr Relat Cancer.

[9] Zhang L, Huang J, Yang N (2006). microRNAs exhibit high frequency genomic alterations in human cancer. Proc Natl Acad Sci USA.

[10] Yang H, Kong W, He L (2008). MicroRNA expression profiling in human ovarian cancer: miR-214 induces cell survival and cisplatin resistance by targeting PTEN. Cancer Res.

[11] Wyman SK, Parkin RK, Mitchell PS, Fritz BR (2009). Repertoire of microRNAs in epithelial ovarian cancer as determined by next generation sequencing of small RNA cDNA libraries. PLoS One.

[12] Wang R, Wang ZX, Yang JS, Pan X, De W, Chen LB (2011). MicroRNA-451 functions as a tumor suppressor in human non-small cell lung cancer by targeting ras-related protein 14 (RAB14). Oncogene.

[13] Rui W, Bing F, Hai-Zhu S, Wei D, Long-Bang C (2010). Identification of microRNA profiles in docetaxel-resistant human non-small cell lung carcinoma cells (SPC-A1). J Cell Mol Med.

[14] Bovicelli A, D’Andrilli G, Giordano A (2011). New players in ovarian cancer. J Cell Physiol.

[15] Li SD, Zhang JR, Wang YQ, Wan XP (2010). The role of microRNAs in ovarian cancer initiation and progression. J Cell Mol Med.

[16] Lima RT, Busacca S, Almeida GM, Gaudino G, Fennell DA, Vasconcelos MH (2011). MicroRNA regulation of core apoptosis pathways in cancer. Eur J Cancer.

[17] Dykxhoorn DM (2010). MicroRNAs and metastasis: little RNAs go a long way. Cancer Res.

[18] van Jaarsveld MT, Helleman J, Berns EM, Wiemer EA (2010). MicroRNAs in ovarian cancer biology and therapy resistance. Int J Biochem Cell Biol.

[19] Lu L, Katsaros D, de la Longrais IA, Sochirca O, Yu H (2007). Hypermethylation of let-7a-3 in epithelial ovarian cancer is associated with low insulin-like growth factor-II expression and favorable prognosis. Cancer Res.

[20] Lu L, Schwartz P, Scarampi L (2011). MicroRNA let-7a: a potential marker for selection of paclitaxel in ovarian cancer management. Gynecol Oncol.

[21] Lou Y, Yang X, Wang F, Cui Z, Huang Y (2010). MicroRNA-21 promotes the cell proliferation, invasion and migration abilities in ovarian epithelial carcinomas through inhibiting the expression of PTEN protein. Int J Mol Med.

[22] Yang H, Kong W, He L (2008). MicroRNA expression profiling in human ovarian cancer: miR-214 induces cell survival and cisplatin resistance by targeting PTEN. Cancer Res.

[23] Sorrentino A, Liu CG, Addario A, Peschle C, Scambia G, Ferlini C (2008). Role of microRNAs in drug-resistant ovarian cancer cells. Gynecol Oncol.

[24] Li Z, Hu S, Wang J (2010). MiR-27a modulates MDR1/P-glycoprotein expression by targeting HIPK2 in human ovarian cancer cells. Gynecol Oncol.

[25] Shi W, Alajez NM, Bastianutto C (2010). Significance of Plk1 regulation by miR-100 in human nasopharyngeal cancer. Int J Cancer.

[26] Leite KR, Tomiyama A, Reis ST (2011). MicroRNA-100 expression is independently related to biochemical recurrence of prostate cancer. J Urol.

[27] Zheng YS, Zhang H, Zhang XJ (2011). MiR-100 regulates cell differentiation and survival by targeting RBSP3, a phosphatase-like tumor suppressor in acute myeloid leukemia. Oncogene.

[28] Takaki T, Trenz K, Costanzo V, Petronczki M (2008). Polo-like kinase 1 reaches beyond mitosis-cytokinesis, DNA damage response, and development. Curr Opin Cell Biol.

[29] Weichert W, Denkert C, Schmidt M (2004). Polo-like kinase isoform expression is a prognostic factor in ovarian carcinoma. Br J Cancer.

[30] Degenhardt Y, Lampkin T (2010). Targeting Polo-like kinase in cancer therapy. Clin Cancer Res.

[31] Strebhardt K, Ullrich A (2006). Targeting polo-like kinase 1 for cancer therapy. Nat Rev Cancer.

[32] Gao Q, Huang X, Tang D (2006). Influence of chk1 and plk1 silencing on radiation- or cisplatin-induced cytotoxicity in human malignant cells. Apoptosis.

